# Blood flow restriction accelerates aerobic training-induced adaptation of ${\dot{\text{V}}}{\text{O}}_{2}$ kinetics at the onset of moderate-intensity exercise

**DOI:** 10.1038/s41598-022-22852-3

**Published:** 2022-10-28

**Authors:** Amane Hori, Ryuji Saito, Kenichi Suijo, Michael R. Kushnick, Daisuke Hasegawa, Koji Ishida, Norio Hotta

**Affiliations:** 1 Graduate School of Life and Health Sciences, Chubu University, Kasugai, Japan; 2 Japan Society for the Promotion of Science, Tokyo, Japan; 3 College of Life and Health Sciences, Chubu University, Kasugai, Japan; 4 College of Health and Human Sciences, Northern Illinois University, DeKalb, IL USA; 5 Research Center of Health, Physical Fitness and Sports, Nagoya University, Nagoya, Japan

**Keywords:** Respiration, Blood flow

## Abstract

It is unclear whether blood flow restriction (BFR) accelerates the adaptation of the time constant (τ) of phase II oxygen uptake (${\dot{\text{V}}}{\text{O}}_{2}$) kinetics in the moderate-intensity exercise domain via moderate-intensity aerobic training. Therefore, healthy participants underwent moderate-intensity [45–60% ${\dot{\text{V}}}{\text{O}}_{2}$ Reserve] aerobic cycle training with or without BFR (BFR group, n = 9; CON group, n = 9) for 8 weeks to evaluate ${\dot{\text{V}}}{\text{O}}_{2}$ kinetics during moderate-intensity cycle exercise before (Pre) and after 4 (Mid) and 8 (Post) weeks of training. Both groups trained for 30 min, 3 days weekly. BFR was performed for 5 min every 10 min by applying cuffs to the upper thighs. The τ significantly decreased by Mid in the BFR group (23.7 ± 2.9 s [Pre], 15.3 ± 1.8 s [Mid], 15.5 ± 1.4 s [Post], *P* < 0.01) and by Post in the CON group (27.5 ± 2.0 s [Pre], 22.1 ± 0.7 s [Mid], 18.5 ± 1.9 s [Post], *P* < 0.01). Notably, the BFR group’s τ was significantly lower than that of the CON group at Mid (*P* < 0.01) but not at Post. In conclusion, BFR accelerates the adaptation of the ${\dot{\text{V}}}{\text{O}}_{2}$ kinetics of phase II by moderate-intensity aerobic training.

## Introduction

At the onset of light-to-moderate (below lactate threshold [LT]) step load exercise, oxygen uptake (${\dot{\text{V}}}{\text{O}}_{2}$) measured at the pulmonary level increases abruptly during the first 15–20 s (phase I or cardio-dynamic phase)^1^. Next, ${\dot{\text{V}}}{\text{O}}_{2}$ increases mono-exponentially (phase II) to a steady-state (phase III)^1^. In healthy individuals performing upright moderate-intensity cycle exercise, the kinetics of pulmonary ${\dot{\text{V}}}{\text{O}}_{2}$ in phase II, identified as the time constant (τ) calculated from model fitting, corresponds to changes in the oxidative metabolism of exercising muscles^2–8^.

Acceleration of phase II τ could contribute to a decrease in the accumulation of hydrogen ion by reducing O_2_ deficit at the onset of exercise, resulting in reduced exercise-induced muscle fatigue^9^. Furthermore, phase II τ is affected by health conditions^10^. For instance, phase II τ was reported to be slowed by aging and some diseases (e.g., chronic heart failure and chronic obstructive pulmonary disease)^10^. Therefore, kinetic analysis of ${\dot{\text{V}}}{\text{O}}_{2}$, especially the evaluation of phase II τ, is useful from exercise performance and clinical perspectives.

The blood flow restriction (BFR) technique is an adjunctive approach to the strength training initially proposed by Sato^11^. It has been reported to elicit a significant increase in muscle size and musculoskeletal function even when relatively low training loads (e.g., 15–30% of 1-repetition maximum) are used^12^. In addition to musculoskeletal adaptations^13–17^, it has been reported that the BFR technique increases maximal oxygen uptake (${\dot{\text{V}}}{\text{O}}_{{2\max }}$) when it is added to low-intensity aerobic exercise^13,18–20^.

Aerobic training has been shown to reduce phase II τ in the moderate-intensity domain^21^, suggesting that the O_2_ deficit at the onset of the same-intensity exercise is reduced^22^. Previous investigations revealed that applying BFR enhances aerobic training-induced increase in muscular oxidative capacity^13,18^. Corvino et al.^23^ reported that 4-week low-intensity (30% of peak power) intermittent aerobic training with BFR reduced phase II τ during the moderate-intensity exercise.

Although low-intensity aerobic training does not alter phase II τ at the onset of exercise^23^, moderate-intensity aerobic training (the American College of Sports Medicine^24^ defines 40–59% ${\dot{\text{V}}}{\text{O}}_{2}$ reserve [${\dot{\text{V}}}{\text{O}}_{2} {\text{R}}$] as the moderate-intensity) without BFR has been reported to improve phase II τ^21^. For instance, Berger et al.^21^ showed that in healthy participants, 6 weeks of 30-min aerobic training improved phase II τ in the moderate-intensity domain. However, no studies have investigated the ${\dot{\text{V}}}{\text{O}}_{2}$ kinetics seen in the on-transient of the moderate-intensity domain when BFR is applied to moderate-intensity aerobic training.

Since previous studies suggested that BFR training increases muscle oxidative capacity^23,25^, application of BFR can further lower moderate-intensity aerobic training-induced decline in phase II τ. However, there could be a possible upper limit where phase II τ is not further improved by aerobic training^26–28^. Hence, we hypothesized that the combination of moderate-intensity aerobic training and BFR would accelerate the adaptation of phase II τ rather than further lower phase II τ. Therefore, this study aimed to investigate the effect of moderate-intensity (45–60% ${\dot{\text{V}}}{\text{O}}_{2} {\text{R}}$) aerobic cycle training with BFR on ${\dot{\text{V}}}{\text{O}}_{2}$ kinetics at the onset of moderate-intensity cycle exercise. To test the hypothesis, the duration of the training was 8 weeks, twice as long as in the previous study^23^, with physiological measurements taken pre- (Pre), mid- (Mid), and post-training (Post).

## Materials and methods

### Design and procedure

The participants were randomly divided into two training groups: an 8-week aerobic cycle training with BFR (BFR group) and without BFR (CON group). The training requirements were the same for each group, and between-group parameters were compared to evaluate the effect of BFR.

**Figure 1:**
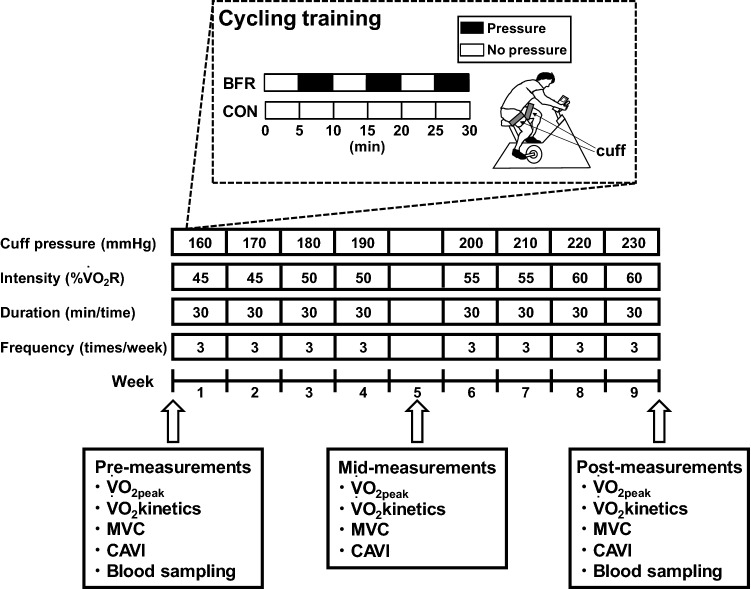
Schema of the training protocol and measurements The participants were randomly assigned to either group: 8-week aerobic cycle training with (BFR group) or without (CON group) blood flow restriction (BFR) and trained for 30 min, 3 days a week for 8 weeks. Cycle training did not occur during the fifth week, and mid-measurements were collected. In the BFR group, cuffs were worn on the upper thighs, and pressure was applied for 5 min every 10 min of exercise. Peak oxygen uptake (${\dot{\text{V}}}{\text{O}}  {{2{\text{peak}}}}$), oxygen uptake (${\dot{\text{V}}}{\text{O}}_{2}$) kinetic parameters during moderate-intensity constant-load exercise, maximal voluntary contraction (MVC) force in knee extensors, and cardio-ankle vascular index (CAVI) were assessed before (Pre) and after 4 (Mid) and 8 (Post) weeks training. In addition, blood from each participant was sampled in Pre and Post to evaluate markers of coagulation and fibrinolysis.

Initially, preliminary testing was conducted to familiarize all participants with the equipment and experimental procedures. Physiological measurements were taken at Pre (on a day different from the preliminary test), Mid (within 1 week after the end of the fourth week), and Post (within 1 week after the end of the eighth week) (Fig. 1).

### Participants and ethical approval

An a priori statistical power analysis was performed to determine the sample size required for this study using G*power 3.1.9.7 (Institute of Experimental Psychology, Heinrich Heine University, Dusseldorf, Germany). The main outcome of this study was the reduction in τ of the primary component of pulmonary ${\dot{\text{V}}}{\text{O}}_{2}$ kinetics. Based on data from a previous study, in which BFR aerobic training induced a significantly greater reduction in τ of the primary component of ${\dot{\text{V}}}{\text{O}}_{2}$ kinetics than that of the control group^23^, the effect size was set to medium. A minimum total sample size of 14 was determined to achieve power (1–β) of more than 0.80, which was required to reject the null hypothesis, with a medium effect size (partial eta squared [η_p_^2^] = 0.06) and an error probability of 0.05 (α), using a repeated measure two-way analysis of variance (ANOVA) (number of groups = 2, number of measurements = 3). The correlation among repeated measurements was set at 0.79 based on a previous study^29^.

Seventeen men and one woman volunteered to participate in this study and were divided into BFR (n = 9, age, 22 ± 2 yr; height, 175.2 ± 2.1 cm; weight, 71.2 ± 3.1 kg) and CON groups (n = 9, age, 21 ± 1 yr; height, 173.8 ± 2.3 cm; weight, 66.7 ± 3.1 kg). All participants were required to be non-smokers, free of chronic diseases, and on no medication. The random grouping assigned the female participant to the CON group. The participants in this study led active lifestyles; eight individuals belonged to athletic clubs but were distributed between both groups (n = 4 in the BFR group, n = 4 in the CON group). All participants did not change their physical activity or exercise patterns during the study besides what was prescribed for the cycle training.

The Ethics Committee of Chubu University approved this study (Approval No.: 240005), and all procedures were conducted following approved institutional guidelines and regulations. The participants were informed of the experimental protocol, including the benefits and risks. All participants provided written informed consent.

### Measurements

Pre-, Mid-, and Post-measurements were performed after at least 2 h of fasting over 2 days. All participants were prohibited from heavy exercise and alcohol and caffeine consumption the day before the measurement. The blood for evaluating blood coagulation or fibrinolysis was collected only Pre and Post (Fig. 1).

#### Cardiorespiratory fitness

After 5 min of upright seated rest and a subsequent three min of unloaded cycling, the participants performed an incremental exercise test of 20 W/min using an electromagnetically braked cycle ergometer (Aerobike 75XLIII, COMBI wellness, Tokyo, Japan) at 60 rpm to determine each individual's peak oxygen uptake (${\dot{\text{V}}}{\text{O}}  {{2{\text{peak}}}}$), defined as the highest 20 s averaged value during the test. The saddle position was adjusted for each participant and participants were retained in that position for each testing and training session. The pedaling rate was kept constant at 60 rpm using a metronome. The test was terminated if the participant could not maintain over 50 rpm, and the cadence was returned to 60 rpm regardless of the experimenters’ verbal exhortation. A breath-by-breath metabolic measurement system (Aeromonitor AE-310 s, Minato Medical Science, Osaka, Japan) measured respiratory parameters and heart rate (HR). The breath-by-breath pulmonary gas exchange was assessed at the mouth level.

In the Pre-test, ${\dot{\text{V}}}{\text{O}}_{2}$ at the gas exchange threshold (GET) was estimated as the breakpoint in the plot of carbon dioxide output against a function of ${\dot{\text{V}}}{\text{O}}_{2}$ (V-slope method)^30^ to determine the intensity of exercise for evaluation of ${\dot{\text{V}}}{\text{O}}_{2}$ kinetics. When determining the GET using the V-slope method was not feasible, it was determined using the ventilatory equivalent method^31^.

#### Muscle strength

Isometric maximal voluntary contraction (MVC) torque produced by single knee extensors was measured using a dynamometer (T.K.K.5402, Takei Science Instrument, Nagoya, Japan) attached to an experimental chair (T.K.K.5715, Takei Science Instrument), based on a previous study^32^. The participants performed three maximal efforts while seated with their knee at approximately 100° (180° = full knee extension), separated by approximately 60-s rest intervals, and were verbally encouraged to exert MVC. The highest torque was recorded, and the torques for both legs were averaged.

#### Arterial stiffness and blood coagulation/fibrinolysis

The participants’ overall arterial stiffness and blood coagulation or fibrinolysis-associated factors were measured to evaluate the safety of the cycle training with BFR in this study. We measured the cardio-ankle vascular index (CAVI)^33^ to evaluate arterial stiffness using a semi-automated vascular screening system (Vasera, 1500 N, Fukuda Denshi, Tokyo, Japan) with the participant in the supine position after supine rest for 10–15 min.

Approximately 5 mL of blood was drawn from an antecubital vein at Pre and Post. Blood was collected in tubes treated with 3.2% sodium citrate (0.5 mL) and centrifuged at 3000 rpm for 15 min at 4 °C to obtain the plasma. Plasma samples were stored at − 80 °C until analysis in a single plasma thrombin/antithrombin III complex (TAT), tissue plasminogen activator/plasminogen activator inhibitor 1 complex (t-PAIC), and D-dimer levels, which were measured at a commercially available laboratory (SRL, Nagoya, Japan).

#### ${\dot{\text{V}}}{\text{O}}_{2}$ during moderate-intensity exercise and ${\dot{\text{V}}}{\text{O}}_{2}$ kinetics analysis

The same cycle ergometer and metabolic measurement system to assess ${\dot{\text{V}}}{\text{O}}  {{2{\text{peak}}}}$, as described above, were used. Each participant performed a moderate-intensity constant-load cycle exercise on a separate day from the incremental exercise test. The participants performed the transition from rest to 5 min of exercise^34^, with the intensity set at 90% GET determined at Pre^35^. As described in a previous study, the exercise test was repeated thrice with intervals between sets until the HR returned to within five bpm in the resting level before the exercise^36^.

**Figure 2:**
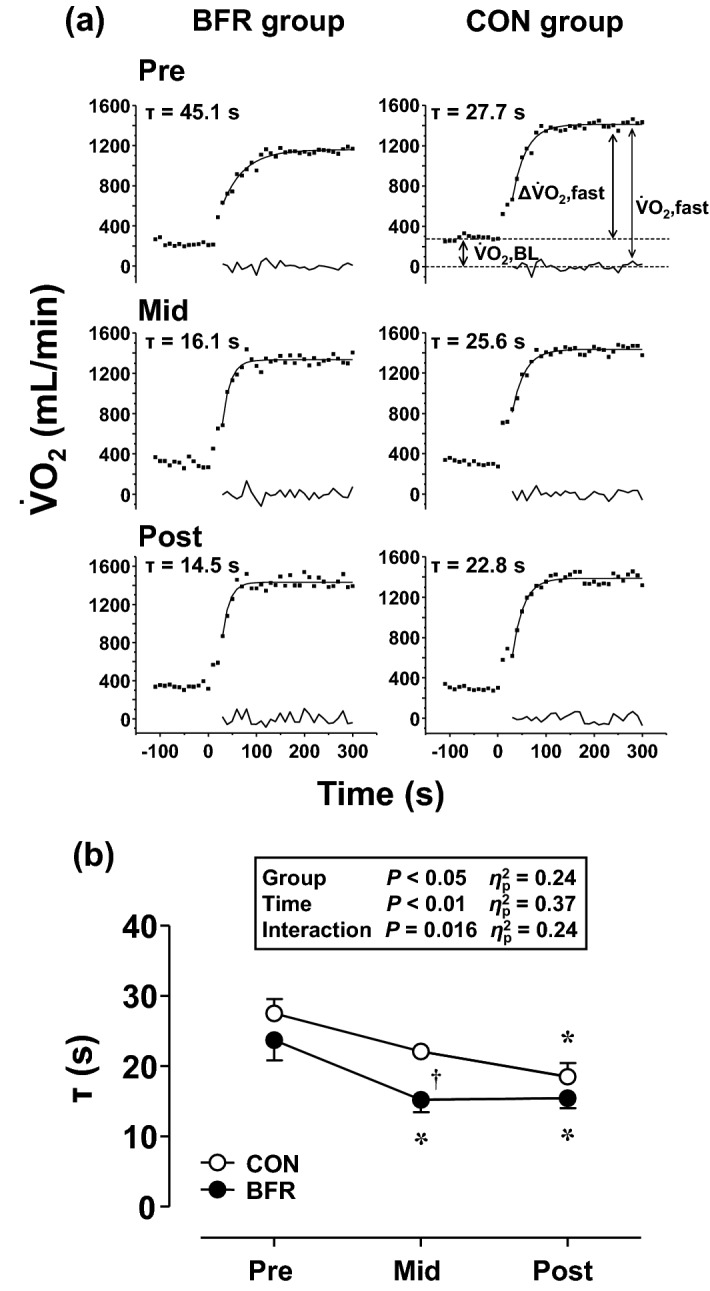
Representative oxygen uptake (${\dot{\text{V}}}{\text{O}}_{2}$) response to moderate-intensity constant-load exercise (**a**) and group means of time constant (τ) of the fast component during moderate-intensity constant-load exercise (**b**) before (Pre), 4 weeks after (Mid), and 8 weeks after (Post) cycle training with (BFR group) or without (CON group) blood flow restriction (BFR) (**a**): ${\dot{\text{V}}}{\text{O}}_{2}$, BL, the baseline of ${\dot{\text{V}}}{\text{O}}_{2}$; $\Delta {\dot{\text{V}}}{\text{O}}  {2}$, fast, the asymptotic amplitude of the fast component (the increase in ${\dot{\text{V}}}{\text{O}}_{2}$ from the baseline to the asymptote of the fast component); ${\dot{\text{V}}}{\text{O}}_{2}$, fast, the absolute asymptotic amplitude of the fast component (the sum of the baseline and the asymptotic amplitude). (**b**): Values are expressed as means ± SE. Statistical analysis was performed using a repeated-measures two-way analysis of covariance, followed by Bonferroni's multiple comparisons test. Partial eta squared (η_p_^2^) is the effect size of the interaction and main effects of group and time. *Significant difference from Pre (*P* < 0.01). †Significant difference between BFR and CON groups at that time point (*P* < 0.01).

For kinetics analysis, any breaths more than four standard deviations away from the local mean of the surrounding six breaths were excluded to remove non-physiological data points of the respiratory parameters^4^. The breath-by-breath ${\dot{\text{V}}}{\text{O}}_{2}$ data were aligned with the onset of exercise and then linearly interpolated between each breath to yield data points at 1-s intervals^4^. Ensemble averaging was performed, and the data were averaged every 10 s to reduce breath-to-breath noise^4^. The resultant ${\dot{\text{V}}}{\text{O}}_{2}$ response was modeled with nonlinear least-squares fitting procedures to an exponential response using Origin 6.1 computer software (Origin Lab, Northampton, MA, USA). The on-transit of ${\dot{\text{V}}}{\text{O}}_{2}$ response to exercise was modeled as a mono-exponential, beginning after the initial component (phase I) for 20 s^7,21,37^ and continuing to a steady-state of the fast component (phase II)^1^:

$${\dot{\text{V}}\text{O}}_{{2}} \left( {\text{t}} \right) \, = {\dot{\text{V}}\text{O}}_{{2}} ,{\text{BL }} + \, \Delta {\dot{\text{V}}\text{O}}_{{2}} ,{\text{fast }}({1}{-}{\text{e}}^{{ - \left( {{\text{t}}{-}{\text{TD}}} \right)/\tau }} )$$

where ${\dot{\text{V}}}{\text{O}}_{2}$,BL is the baseline, $\Delta {\dot{\text{V}}}{\text{O}}  {2}$,fast is the asymptotic amplitude to which ${\dot{\text{V}}}{\text{O}}_{2}$ projects (Fig. 2a), τ is the time constant of the response, and TD is the delay time. In the fast component, the asymptote, i.e., absolute amplitude (${\dot{\text{V}}}{\text{O}}_{2}$,fast), is demonstrated as the sum of ${\dot{\text{V}}}{\text{O}}_{2}$,BL and $\Delta {\dot{\text{V}}}{\text{O}}  {2}$,fast (Fig. 2a).

### Training protocol

The training requirements are shown in Fig. 1. Both groups participated in the cycle training program using a cycle ergometer with the pedal cadence set at 60 rpm. The participants were trained for 30 min per session, 3 days per week for 8 weeks. The training was not performed during the fifth week due to Mid assessments. Training frequency and duration were based on a previous study in which 15 min of cycle training with BFR at 40% ${\dot{\text{V}}}{\text{O}}_{{2\max }}$ for 8 weeks increased ${\dot{\text{V}}}{\text{O}}_{{2\max }}$ but did not achieve a significant increase in muscle strength. Also, 30 min of cycle training with BFR at the same 40% ${\dot{\text{V}}}{\text{O}}_{{2\max }}$ for 8 weeks increased muscle strength^13^. Therefore, the duration of each exercise session was set at 30 min to promote an increase in muscle strength^38^. For the first and second weeks, the training sessions were performed at 45% ${\dot{\text{V}}}{\text{O}}_{2} {\text{R}}$, based on a review by Swain and Franklin^39^. They reported this value as the minimal effective training intensity for higher fitness participants to elicit changes in ${\dot{\text{V}}}{\text{O}}_{{2\max }}$. ${\dot{\text{V}}}{\text{O}}_{2} {\text{R}}$ was calculated from the incremental exercise test performed at Pre and progressively increased by 5% ${\dot{\text{V}}}{\text{O}}_{2} {\text{R}}$ every 2 weeks, ending at 60% ${\dot{\text{V}}}{\text{O}}_{2} {\text{R}}$. The participants were required to drink at least 500 mL of cold water during and/or after each training session to reduce the risk of venous thrombosis.

For each training session, the participants in the BFR group wore a 60-mm wide cuff (SC5, D.E. Hokanson, WA, USA) connected to a cuff inflator (Occluder, ARCOSYSTEM, Japan) around each thigh approximately 1–2 cm distal to the inguinal folds^40^. A cuff pressure of 160–240 mmHg has been suggested to be appropriate for most individuals in terms of the efficacy and safety if 6 cm cuffs are used for the lower body^41^. In addition, a previous study has reported increased aerobic capacity following aerobic cycle training with BFR, in which the cuff pressure was set at 160–210 mmHg^13^. Restriction pressures in this range have been suggested to reduce venous blood flow and cause blood pooling in capacitance vessels distal to the tourniquet cuff^42,43^. Therefore, we utilized this range of cuff pressures in this study. The cuff pressure was rapidly inflated to 160 mmHg 5 min after initiation of exercise in the first week. It was progressively increased by 10 mmHg each week until a final cuff pressure of 230 mmHg was reached in the eighth week of training (Fig. 1). The BFR pressure was maintained for 5 min interspersed with no pressure for 5 min during each training session to enhance safety^44^; therefore, each training session included 15 min with pressure and 15 min without pressure.

Since the cardiovascular safety of BFR has been reported as a concern^42,45,46^, we evaluated the effects of BFR on cardiovascular response and rating of perceived exertion (RPE) using the Borg scale^47^ during the cycle training sessions. During the cycle training with BFR, HR was measured using an electrocardiogram (ECG; Tango + , Sun Tech Medical, NC, USA), and both systolic and diastolic blood pressure (SBP and DBP, respectively) were determined using an electro-sphygmomanometer (Tango +) in the upper arm. These parameters were measured before the start of exercise and every five min during exercise once per week in the BFR group.

### Statistical analysis

The Shapiro–Wilk test was performed to confirm data normality. An unpaired *t*-test or Mann–Whitney U test and a chi-square test were used for between-group comparisons and ratio comparisons, respectively. Cohen's *d* was used for the between-group comparison to determine the effect sizes. ${\dot{\text{V}}}{\text{O}}_{2}$ kinetic parameters, aerobic capacity, muscle strength, arterial stiffness, and blood coagulation or fibrinolysis were analyzed using a repeated-measures two-way analysis of covariance. Factors included “group” (BFR and CON groups) and “time” (Pre, Mid, and Post), and the parameters were adjusted using each Pre value (baseline) as a covariate to account for the influence of corresponding changes in those parameters. If a significant group × time interaction or the main effect was detected, we identified the specific differences using Bonferroni's multiple comparisons test. Furthermore, the effect sizes for the main effects of group and time and the interaction by calculating the η_p_^2^ were determined. A repeated-measures one-way ANOVA or Friedman test was utilized for the HR, SBP, and DBP achieved during the cycle training with BFR. When a repeated-measures one-way ANOVA or Friedman test showed significance, we performed Bonferroni's or Dunn's multiple comparisons test. Effect sizes for one-way ANOVA or Friedman test were determined by η_p_^2^ or Kendall's W, respectively. Statistical analyses were computed using SPSS 25.0 (IBM, Armonk, NY, USA), and the significance level was set at 0.05. Values are expressed as the mean ± SE.

## Results

There were no significant differences regarding age, body weight, or height between the groups (*P* > 0.48, d ≤ 0.49). All participants completed all testing and training sessions. No significant between-group differences were detected at Pre in ${\dot{\text{V}}}{\text{O}}_{2}$ kinetic parameters at the onset of exercise (*P* > 0.11, d < 0.59), ${\dot{\text{V}}}{\text{O}}  {{2{\text{peak}}}}$ (*P* = 0.53, d = 0.31), MVC torque (*P* = 0.14, d = 0.73), CAVI (*P* = 0.63, d = 0.23), or in any blood coagulation or fibrinolysis parameters (*P* > 0.59, d < 0.26).

### Cardiovascular response during cycle exercise with BFR

HR and SBP significantly increased above resting values during the training session (Supplementary Table 1). BP responses and RPE significantly increased as pressure was applied during the training sessions; only BP responses significantly declined once the BFR was removed from the thigh cuffs at some time points (Supplementary Table 1).

### ${\dot{\text{V}}}{\text{O}}_{2}$ kinetics

Figure 2a shows a typical ${\dot{\text{V}}}{\text{O}}_{2}$ response during moderate-intensity exercise. As shown in Table 1, $\Delta {\dot{\text{V}}}{\text{O}}  {2}$, fast was not significantly altered by training with or without BFR. Figure 2b shows the changes in the τ. The aerobic training significantly decreased the τ at Mid in the BFR group but not in the CON group (*P* = 0.054). The τ at Post were significantly lower than those at Pre in the BFR and CON groups. These significant changes from Pre to Mid (Δ8.5 ± 3.3 s) and Pre to Post (Δ8.3 ± 3.2 s) in the BFR group and from Pre to Post (Δ9.0 ± 1.2 s) in the CON group were higher than the 95% confidence intervals, which reflects a confidence in the model fit and the estimation of the model parameters (τ) describing this fit (Table 1). Notably, the interaction was significant. Furthermore, the τ at Mid in the BFR group was significantly lower than that in the CON group; however, there was no significant difference between groups at Post (*P* = 0.39).

**Table 1:** Oxygen uptake (${\dot{\text{V}}}{\text{O}}_{2}$) kinetic parameters during moderate-intensity constant-load exercise before (Pre), 4 weeks after (Mid), and 8 weeks after (Post) cycle training with (BFR group) or without (CON group) blood flow restriction (BFR).

			Pre			Mid				Post				Main effect		Interaction
														Time	Group	
${\dot{\text{V}}}{\text{O}}_{2}$,BL	(mL/min)	BFR	315.05	±	13.76	325.23	±	12.92		323.43	±	13.04		*P* < 0.01 η_p_^2^ = 0.28	*P* = 0.10 η_p_^2^ = 0.17	*P* = 0.25 η_p_^2^ = 0.09
		CON	317.70	±	13.79	311.09	±	9.28		295.47	±	12.12				
$\Delta{\dot{\text{V}}}{\text{O}}{2}$,fast	(mL/min)	BFR	1084.66	±	30.71	1045.53	±	36.17		1063.13	±	32.29		*P* = 0.14 η_p_^2^ = 0.13	*P* = 0.46 η_p_^2^ = 0.04	*P* = 0.57 η_p_^2^ = 0.03
		CON	1151.77	±	44.89	1072.19	±	45.02		1074.56	±	37.71				
${\dot{\text{V}}}{\text{O}}_{2}$,fast	(mL/min)	BFR	1399.71	±	42.62	1370.77	±	46.25		1386.56	±	33.22		*P* < 0.05 η_p_^2^ = 0.21	*P* = 0.38 η_p_^2^ = 0.05	*P* = 0.42 η_p_^2^ = 0.05
		CON	1469.47	±	50.92	1383.28	±	49.32		1370.03	±	45.54				
TD	(s)	BFR	7.52	±	6.22	18.35	±	1.40	*	18.50	±	1.26	*	*P* < 0.01 η_p_^2^ = 0.88	*P* = 0.24 η_p_^2^ = 0.09	*P* = 0.25 η_p_^2^ = 0.09
		CON	12.08	±	2.24	14.83	±	1.02		18.01	±	1.94				
95%CI for τ	(s)	BFR	6.88	±	0.71	6.65	±	0.91		6.74	±	0.52		*P* = 0.18 η_p_^2^ = 0.108	*P* = 0.78 η_p_^2^ = 0.005	*P* = 0.96 η_p_^2^ = 0.003
		CON	6.93	±	0.52	6.50	±	0.60		6.44	±	0.89				

${\dot{\text{V}}}{\text{O}}_{2}$,BL, baseline of ${\dot{\text{V}}}{\text{O}}_{2}$; $\Delta {\dot{\text{V}}}{\text{O}}  {2}$,fast, asymptotic amplitude of the fast component (the increase in ${\dot{\text{V}}}{\text{O}}_{2}$ from the baseline to the asymptote of the fast component); ${\dot{\text{V}}}{\text{O}}_{2}$,fast, absolute asymptotic amplitude of the fast component (the sum of the baseline and the asymptotic amplitude); TD, delay time; 95% CI for τ, 95% confidence interval for phase II time constant. Statistical analysis was performed using a repeated-measures two-way analysis of covariance followed by Bonferroni's multiple comparisons test. Partial eta squared (η_p_^2^) is the effect size of the interaction and main effects of group and time. Values are expressed as mean ± SE (n = 9).

*Significant difference from pre-training (pre) (*P* < 0.05).

### Cardiorespiratory fitness, muscle strength, arterial stiffness, and blood coagulation or fibrinolysis-associated factors

Table 2 presents the changes from Pre to Post in ${\dot{\text{V}}}{\text{O}}  {{2{\text{peak}}}}$, MVC torque, and CAVI. ${\dot{\text{V}}}{\text{O}}  {{2{\text{peak}}}}$ and peak load during the incremental exercise test significantly increased using cycle exercise training with and without BFR. The peak load at Mid in the BFR group was significantly higher than that in the CON group. In contrast, ${\dot{\text{V}}}{\text{O}}  {{2{\text{peak}}}}$ at Mid and Post was not significantly different between groups. The exercise training significantly increased the MVC torque in the BFR group but not in the CON group (*P* > 0.99) at Post. Furthermore, MVC torque at Post in the BFR group was significantly higher than that in the CON group. There were no significant differences in CAVI between the groups; training or BFR did not significantly alter CAVI either.

**Table 2:** Changes in peak oxygen uptake (${\dot{\text{V}}}{\text{O}}  {{2{\text{peak}}}}$), peak load, maximal voluntary contraction (MVC) torque in knee extensors, and cardio-ankle vascular index (CAVI) before (Pre), 4 weeks after (Mid), and 8 weeks after (Post) cycle training with (BFR group) or without (CON group) blood flow restriction (BFR).

			Pre			Mid					Post					Main effect		Interaction
																Time	Group	
${\dot{\text{V}}}{\text{O}}_2 {\text{peak}}$	(mL/kg/min)	BFR	42.4	±	1.4	45.8	±	1.7	*		48.4	±	1.8	*		*P* < 0.05 η_p_^2^ = 0.25	*P* = 0.48 η_p_^2^ = 0.03	*P* = 0.599 η_p_^2^ = 0.03
		CON	40.5	±	2.6	43.3	±	2.5	*		45.6	±	2.2	*				
Peak load	(W)	BFR	256.7	±	6.0	290.1	±	8.4	*	†	307.9	±	8.6	*		*P* < 0.01 η_p_^2^ = 0.31	*P* < 0.05 η_p_^2^ = 0.24	*P* < 0.05 η_p_^2^ = 0.20
		CON	236.9	±	14.0	259.1	±	11.8	*		277.6	±	12.0	*				
MVC	(N･m)	BFR	292.4	±	11.8	312.8	±	17.4			337.4	±	20.8	*	†	*P* = 0.29 η_p_^2^ = 0.08	*P* < 0.05 η_p_^2^ = 0.27	*P* < 0.01 η_p_^2^ = 0.32
		CON	253.5	±	22.4	249.2	±	26.3			247.4	±	20.4					
CAVI	(a.u.)	BFR	5.62	±	0.25	5.48	±	0.24			5.62	±	0.20			*P* < 0.01 η_p_^2^ = 0.28	*P* = 0.13 η_p_^2^ = 0.15	*P* = 0.36 η_p_^2^ = 0.07
		CON	5.81	±	0.29	5.90	±	0.20			5.92	±	0.19					

Statistical analysis was performed using a repeated-measures two-way analysis of covariance followed by Bonferroni's multiple comparisons test. Partial eta squared (η_p_^2^) is the effect size of the interaction and main effects of group and time. Values are expressed as mean ± SE (n = 9).

*Significant difference from pre-training (Pre) (*P* < 0.05). †Significant difference between BFR group and CON group at that time point (*P* < 0.05).

Table 3 shows that there were no significant effects of BFR cycle training on coagulation and fibrinolytic variables. While TAT and t-PAIC did not significantly change from Pre- to Post-training, D-dimer levels significantly decreased from Pre- to Post-training in both groups after 8 weeks of training.

**Table 3:** Changes in thrombin/antithrombin III complex (TAT), tissue plasminogen activator/plasminogen activator inhibitor 1 complex (t-PAIC), and D-dimer before (Pre) and after (Post) 8-week cycle training with (BFR group) or without (CON group) blood flow restriction (BFR).

			Pre			Post				Main effect		Interaction
										Time	Group	
TAT	(ng/mL)	BFR	1.77	±	0.22	2.92	±	1.33		*P* = 0.11 η_p_^2^ = 0.16	*P* = 0.57 η_p_^2^ = 0.02	*P* = 0.57 η_p_^2^ = 0.02
		CON	1.90	±	0.32	2.04	±	0.35				
t-PAIC	(ng/mL)	BFR	21.56	±	2.60	19.78	±	2.72		*P* = 0.15 η_p_^2^ = 0.14	*P* = 0.17 η_p_^2^ = 0.12	*P* = 0.17 η_p_^2^ = 0.12
		CON	24.44	±	4.58	34.67	±	8.94				
D-dimer	(μg/mL)	BFR	0.20	±	0.03	0.13	±	0.01	*	*P* < 0.05 η_p_^2^ = 0.33	*P* = 0.21 η_p_^2^ = 0.10	*P* = 0.21 η_p_^2^ = 0.10
		CON	0.19	±	0.03	0.17	±	0.03				

Statistical analysis was performed using a repeated-measures two-way analysis of covariance followed by Bonferroni's multiple comparisons test. Partial eta squared (η_p_^2^) is the effect size of the interaction and main effects of group and time. Values are expressed as mean ± SE (n = 9).

*Significant difference from pre-training (Pre) (*P* < 0.05).

## Discussion

In the present study, the moderate-intensity aerobic cycle training significantly lowered phase II τ in the initial 4 weeks of training in both the BFR and CON groups. The most important finding was that phase II τ of Mid in the BFR group was significantly lower than that in the CON group. However, no significant difference between the groups was detected at Post. To our knowledge, this study is the first to demonstrate that BFR accelerated the adaptation of the speed of ${\dot{\text{V}}}{\text{O}}_{2}$ kinetics at the onset of moderate-intensity exercise by moderate-intensity constant-load cycle training in healthy young adults.

### Effects of aerobic training with BFR on ${\dot{\text{V}}}{\text{O}}_{2}$ kinetics

As demonstrated in previous studies^21^, this study confirmed that the cycle training sped phase II τ during moderate-intensity exercise. This suggests that the O_2_ deficit at the onset of the same-intensity exercise was reduced^22^. From the performance standpoint, speeding of the τ at moderate intensity could contribute to decrease the accumulation of hydrogen ions from lactic acid at the onset of exercise and thus reduce muscle fatigue^9^. It is assumed that O_2_ extraction is an influential factor in determining phase II τ during upright cycle exercise when performed by healthy individuals^6^. Therefore, aerobic training should have potentiated the capacity for O_2_ extraction in skeletal muscles.

Notably, this study’s adjunctive use of BFR enhanced the aerobic training response during phase II τ of moderate-intensity exercise. Previous studies have demonstrated that acute BFR exercise increased the mRNA levels of the peroxisome proliferator-activated receptor-c co-activator 1α, which stimulates mitochondrial biogenesis^48–50^. In addition, it has previously been reported that 4-week BFR training potentiated citrate synthase activity, a marker of oxidative capacity^25^. Hence, it can be speculated that these adaptations to reduced blood flow and possible transient ischemia during BFR exercise facilitated an increase in oxidative enzymatic activities and O_2_ extraction capacity in skeletal muscles, as this is often cited as part of the explanations for the adaptation of phase II τ.

This study highlights that 4 weeks of BFR aerobic training lowered the ${\dot{\text{V}}}{\text{O}}_{2}$ phase II τ during moderate intensity exercise, as previously reported^23^. However, there was no significant difference in phase II τ between aerobic training with or without BFR at 8 weeks. The effect of aerobic training becomes less pronounced with prolonged training, and there is a point at which no further improvement in phase II τ occurs^26–28^. Indeed, τ at Mid in the present BFR group (approximately 15 s) was close to the smallest value reported in healthy adults^28,37^. Hence, training-induced reduction in ${\dot{\text{V}}}{\text{O}}_{2}$ phase II τ might have reached an upper limit beyond which significant further increases might not occur at 4 weeks of the BFR aerobic training, as discussed in previous studies^23,26–28^. Therefore, the present results suggest that the BFR technique may accelerate the maximal adaptation of phase II τ achieved by moderate-intensity aerobic training.

The present aerobic training significantly increased ${\dot{\text{V}}}{\text{O}}  {{2{\text{peak}}}}$; however, no significant effect of BFR on the adaptation of ${\dot{\text{V}}}{\text{O}}  {{2{\text{peak}}}}$ was detected. This result appears to contradict the above speculation that BFR improves phase II τ since the enhancement of aerobic capacity presumably contributes to an increase in ${\dot{\text{V}}}{\text{O}}  {{2{\text{peak}}}}$^51^. The previous results^52^ demonstrated that the significant association between ${\dot{\text{V}}}{\text{O}}  {{2{\text{peak}}}}$ and phase II τ during the moderate-intensity exercise was observed in untrained individuals but not in trained individuals. Therefore, since some participants in this study were trained individuals, only significant acceleration of phase II τ during moderate-intensity exercise by BFR might be observed.

### Consideration for the application

This study highlights that adding the BFR technique to cycle training may be useful in increasing aerobic function, including ${\dot{\text{V}}}{\text{O}}  {{2{\text{peak}}}}$ and the ${\dot{\text{V}}}{\text{O}}_{2}$ phase II τ, and muscle strength than similar training without BFR, but not without altering arterial stiffness estimated by CAVI, blood coagulation assessed by D-dimer, and fibrinolytic status suggested by TAT and t-PAIC. However, some researchers have reported that BFR may generate an exaggerated reflex-mediated pressor response, which might induce adverse cardiovascular effects during exercise^46,53,54^. In line with other reports, for instance, Renzi et al.^55^, the present supplemental data revealed that the blood pressure response to BFR training might represent a negative aspect of BFR training in some participants. In addition, in contrast to the present study, some studies suggest that BFR aerobic exercise resulted in an increase in arterial stiffness^55,56^. Some researchers indicated that BFR training may have negative effects on coagulation and fibrinolytic status^57,58^. Therefore, although at least the present BFR strategy did not alter arterial stiffness, blood coagulation response, and fibrinolytic status, special consideration would be given to the application of this technique in older adults and patients with cardiovascular diseases.

### Limitations and strengths

This study included some methodological limitations. First, moderate-intensity exercise tests to evaluate ${\dot{\text{V}}}{\text{O}}_{2}$ kinetics were started from rest. This might have affected flywheel inertia. However, this methodological limitation probably did not seriously affect the outcomes in this study because Pre, Mid, and Post tests were performed similarly. Second, evidence suggests that BFR pressure and the cuff size should be individualized per participant for venous pooling without arterial occlusion and safety^12,56,59,60^. However, in this study, we used identical cuff pressure and size for each participant according to a previous study^13^. Therefore, we cannot exclude safety concerns and the possibility that in some participants, the BFR stimulus was not maximum for venous pooling without arterial occlusion. However, both present cuff pressure and size were recommended regarding the efficacy and safety of BFR training^41^. Indeed, many previous studies have demonstrated the benefits of BFR on aerobic exercise effects, although individualized cuff pressure and size were not used (e.g.,^13,16,23,61,62^). Taken together, the cuff pressure and size did not seem to be major problems. Most of participants in this study were male. Therefore, we could not examine the influence of sex on the adaptation of ${\dot{\text{V}}}{\text{O}}_{2}$ kinetics during moderate-intensity exercise by 8-week aerobic BFR training. Despite these limitations, this study has several strengths. We demonstrated that the BFR technique accelerated aerobic training-induced adaptation of phase II τ, thereby suggesting that applying BFR to aerobic training is beneficial for initial training in athletes who may be recovering from injuries or returning from an off-season. In addition, the BFR training regimen in this study improved ${\dot{\text{V}}}{\text{O}}_{2}$ and muscle strength concurrently without significant negative impacts on arterial stiffness and blood coagulation or fibrinolysis status. To our knowledge, no study has shown a concurrent increase in muscle strength and aerobic capacity after moderate-intensity constant-load cycle training with BFR in healthy young adults, without identifying negative effects on blood coagulation or fibrinolysis status and vascular function.

## Conclusion

The results of this study demonstrate that moderate-intensity constant-load cycle training with BFR accelerated the ${\dot{\text{V}}}{\text{O}}_{2}$ kinetics of phase II during the moderate-intensity exercise in healthy young adults.

## Supplementary Information

Supplementary Information.

## Data Availability

The data for this study are available from the corresponding author on reasonable request.
