# Supplementary material for: Blood flow restriction accelerates aerobic training-induced adaptation of V˙O2 kinetics at the onset of moderate-intensity exercise
Source: Sci Rep. 2022 Oct 28;12:18160. doi: 10.1038/s41598-022-22852-3 (PMC9616915; doi:10.1038/s41598-022-22852-3)
Supplement: Supplementary file 1 — Supplementary Information. [file 41598_2022_22852_MOESM1_ESM.pdf]

**Supplementary Table 1. Changes in the systolic and diastolic blood pressures (SBP and DBP), heart rate (HR), and rating of perceived exertion (RPE) during blood flow restricted (BFR) cycling training**

| | | Rest | 5 min | 10 min | 15 min | 20 min | 25 min | 30 min |
|---|---|---|---|---|---|---|---|---|
| | Week | | | BFR | | BFR | | BFR |
| SBP (mmHg) | 1 | 109.9 ± 4.8 | 156.8 ± 7.8 | 168.0 ± 8.5 | 155.7 ± 8.0 | 169.0 ± 8.8 | 153.1 ± 7.7 | 168.6 ± 8.8 |
| | 2 | 111.0 ± 4.0 | 165.0 ± 9.0 | 183.2 ± 11.6 | 163.2 ± 8.9 | 177.4 ± 11.4 | 162.9 ± 11.3 | 180.1 ± 11.4 |
| | 3 | 104.9 ± 3.2 | 172.6 ± 11.5 | 180.8 ± 8.8 | 162.9 ± 9.0 | 180.1 ± 9.8 | 166.4 ± 9.1 | 177.3 ± 9.4 |
| | 4 | 115.6 ± 3.5 | 169.8 ± 5.9 | 181.9 ± 7.8 | 167.6 ± 7.6 | 176.6 ± 8.4 | 160.8 ± 8.1 | 184.4 ± 9.4 |
| | 5 | 117.2 ± 2.1 | 180.9 ± 8.5 | 186.2 ± 9.7 | 174.1 ± 11.6 | 188.9 ± 8.4 | 174.7 ± 8.3 | 189.0 ± 7.5 |
| | 6 | 115.0 ± 3.4 | 161.3 ± 8.2 | 184.8 ± 10.8 | 169.0 ± 10.6 | 187.3 ± 9.9 | 166.2 ± 9.6 | 177.3 ± 9.1 |
| | 7 | 116.8 ± 3.5 | 185.4 ± 9.9 | 191.4 ± 9.0 | 174.1 ± 9.9 | 184.2 ± 8.7 | 169.4 ± 8.6 | 182.1 ± 8.6 |
| | 8 | 113.7 ± 3.7 | 179.6 ± 9.8 | 192.4 ± 10.4 | 176.0 ± 8.4 | 188.7 ± 9.9 | 170.8 ± 10.1 | 185.2 ± 9.1 |
| | 8-week average | 113.0 ± 2.6 | 171.4 ± 7.8 * | 183.6 ± 8.5 * ↑ | 167.8 ± 8.8 * ↓ | 181.5 ± 8.8 * ↑ | 165.5 ± 8.6 * ↓ | 180.5 ± 8.2 * ↑ |
| DBP (mmHg) | 1 | 66.2 ± 3.6 | 61.6 ± 5.1 | 62.1 ± 4.9 | 59.7 ± 4.8 | 64.9 ± 4.2 | 61.8 ± 4.5 | 65.8 ± 5.1 |
| | 2 | 67.2 ± 4.5 | 62.7 ± 3.6 | 69.1 ± 4.5 | 61.7 ± 4.5 | 68.3 ± 4.3 | 61.9 ± 3.6 | 68.7 ± 3.6 |
| | 3 | 65.7 ± 3.3 | 64.3 ± 3.0 | 67.8 ± 3.5 | 63.1 ± 3.4 | 67.0 ± 2.6 | 59.7 ± 3.2 | 64.7 ± 3.1 |
| | 4 | 63.8 ± 4.0 | 57.4 ± 3.8 | 65.8 ± 4.1 | 58.3 ± 4.5 | 65.6 ± 4.4 | 55.6 ± 3.4 | 67.6 ± 3.2 |
| | 5 | 62.7 ± 3.0 | 65.6 ± 3.1 | 71.1 ± 3.9 | 65.7 ± 3.4 | 68.1 ± 5.1 | 63.7 ± 4.4 | 64.8 ± 3.8 |
| | 6 | 70.9 ± 3.2 | 67.8 ± 4.8 | 73.3 ± 6.1 | 63.4 ± 5.3 | 71.1 ± 6.1 | 62.6 ± 4.6 | 68.9 ± 5.1 |
| | 7 | 66.4 ± 4.1 | 75.9 ± 5.5 | 76.8 ± 5.1 | 68.7 ± 5.2 | 71.1 ± 3.8 | 67.9 ± 4.1 | 70.6 ± 4.0 |
| | 8 | 62.9 ± 2.6 | 67.7 ± 3.1 | 71.6 ± 3.8 | 64.3 ± 3.7 | 72.0 ± 3.0 | 66.4 ± 3.4 | 68.8 ± 3.5 |
| | 8-week average | 65.7 ± 2.8 | 65.4 ± 3.5 | 69.7 ± 4.0 | 63.1 ± 3.9 ↓ | 68.5 ± 3.6 ↑ | 62.4 ± 3.3 ↓ | 67.5 ± 3.4 ↑ |
| HR (beats/min) | 1 | 72.9 ± 3.3 | 128.8 ± 4.5 | 137.8 ± 5.1 | 137.9 ± 3.4 | 142.8 ± 5.4 | 135.7 ± 4.3 | 144.6 ± 5.4 |
| | 2 | 72.1 ± 3.4 | 127.7 ± 2.9 | 137.7 ± 4.3 | 135.2 ± 3.6 | 143.9 ± 4.4 | 137.2 ± 3.1 | 144.3 ± 4.9 |
| | 3 | 77.8 ± 3.8 | 138.4 ± 4.5 | 152.4 ± 5.8 | 147.4 ± 5.0 | 158.0 ± 5.7 | 152.1 ± 5.1 | 160.0 ± 6.1 |
| | 4 | 72.1 ± 2.5 | 130.9 ± 2.8 | 142.3 ± 5.0 | 137.4 ± 3.5 | 147.3 ± 4.7 | 139.2 ± 3.6 | 149.9 ± 4.9 |
| | 5 | 70.3 ± 3.2 | 135.2 ± 3.4 | 149.7 ± 4.3 | 143.2 ± 4.6 | 154.7 ± 5.1 | 147.4 ± 4.4 | 160.2 ± 4.4 |
| | 6 | 73.4 ± 2.7 | 131.2 ± 5.6 | 149.0 ± 4.3 | 147.6 ± 4.9 | 159.1 ± 5.3 | 151.1 ± 4.7 | 161.8 ± 5.7 |
| | 7 | 76.0 ± 3.5 | 148.3 ± 3.5 | 162.7 ± 5.3 | 156.7 ± 5.2 | 168.2 ± 6.1 | 162.3 ± 4.9 | 173.0 ± 5.9 |
| | 8 | 72.3 ± 3.3 | 145.2 ± 3.3 | 159.6 ± 4.1 | 155.2 ± 4.4 | 166.7 ± 5.0 | 159.0 ± 4.6 | 168.1 ± 5.0 |
| | 8-week average | 73.4 ± 1.7 | 135.7 ± 2.8 | 148.9 ± 4.3 * | 145.1 ± 3.9 | 155.1 ± 4.8 * | 148.0 ± 3.9 * | 157.7 ± 4.8 * |
| RPE | 1 | | 10.6 ± 0.7 | 12.1 ± 0.8 | 11.2 ± 0.7 | 13.0 ± 0.8 | 11.9 ± 0.8 | 13.4 ± 0.9 |
| | 2 | | 11.1 ± 0.7 | 13.1 ± 0.8 | 11.7 ± 0.7 | 13.9 ± 0.8 | 12.0 ± 0.8 | 14.3 ± 0.9 |
| | 3 | | 11.6 ± 0.6 | 14.2 ± 0.7 | 12.7 ± 0.5 | 14.9 ± 0.4 | 13.2 ± 0.4 | 15.6 ± 0.5 |
| | 4 | | 11.3 ± 0.6 | 14.1 ± 0.5 | 12.4 ± 0.6 | 14.7 ± 0.8 | 13.3 ± 0.7 | 15.1 ± 0.8 |
| | 5 | | 11.8 ± 0.8 | 14.1 ± 0.7 | 13.0 ± 0.8 | 15.1 ± 0.9 | 14.0 ± 1.0 | 15.6 ± 0.9 |
| | 6 | | 11.9 ± 0.5 | 14.4 ± 0.6 | 12.8 ± 0.5 | 15.4 ± 0.6 | 14.0 ± 0.6 | 16.3 ± 0.6 |
| | 7 | | 12.0 ± 0.7 | 14.2 ± 0.8 | 13.1 ± 0.7 | 15.8 ± 0.5 | 14.4 ± 0.6 | 16.8 ± 0.8 |
| | 8 | | 11.9 ± 0.5 | 14.6 ± 0.8 | 13.1 ± 0.6 | 15.4 ± 0.7 | 13.4 ± 0.6 | 16.2 ± 0.8 |
| | 8-week average | | 11.6 ± 0.6 | 14.0 ± 0.6 | 12.6 ± 0.6 | 14.9 ± 0.6 ↑ | 13.4 ± 0.6 | 15.5 ± 0.6 ↑ |

We calculated the 8-week average SBP, DBP, HR, and RPE of each subject at rest, and 5, 10, 15, 20, 25, and 30 min during exercise, for statistical analysis. RPE was evaluated only during exercise. Since a repeated-measures one-way analysis of variance or Friedman test showed significant effects of the training with BFR on all the parameters ($P < 0.05$, partial eta squared > 0.31, Kendall's W > 0.88), Bonferroni's or Dunn's multiple comparisons test was performed. Values are expressed as mean ± SE (n = 9).

*Significant difference compared to the rest. ↑Significant increase from non-BFR to BFR ($P < 0.05$). ↓Significant decrease from BFR to non-BFR ($P < 0.05$).
